# High resolution temperature data for ecological research and management on the Southern Ocean Islands

**DOI:** 10.1038/sdata.2018.177

**Published:** 2018-09-04

**Authors:** Rachel I. Leihy, Grant A. Duffy, Erika Nortje, Steven L. Chown

**Affiliations:** 1 School of Biological Sciences, Monash University, Melbourne, Victoria 3800, Australia; 2 Centre for Invasion Biology, Department of Botany and Zoology, Stellenbosch University, Private Bag X1, Matieland 7602, South Africa

**Keywords:** Conservation biology, Climate-change ecology, Invasive species, Biogeography, Ecological modelling

## Abstract

Southern Ocean Islands are globally significant conservation areas. Predicting how their terrestrial ecosystems will respond to current and forecast climate change is essential for their management and requires high-quality temperature data at fine spatial resolutions. Existing datasets are inadequate for this purpose. Remote-sensed land surface temperature (LST) observations, such as those collected by satellite-mounted spectroradiometers, can provide high-resolution, spatially-continuous data for isolated locations. These methods require a clear sightline to measure surface conditions, however, which can leave large data-gaps in temperature time series. Using a spatio-temporal gap-filling method applied to high-resolution (~1 km) LST observations for 20 Southern Ocean Islands, we compiled a complete monthly temperature dataset for a 15-year period (2001–2015). We validated results using *in situ* measurements of microclimate temperature. Gap-filled temperature observations described the thermal heterogeneity of the region better than existing climatology datasets, particularly for islands with steep elevational gradients and strong prevailing winds. This dataset will be especially useful for terrestrial ecologists, conservation biologists, and for developing island-specific management and mitigation strategies for environmental change.

## Background & Summary

The Southern Ocean Islands (SOIs) are among the most remote islands on Earth. They house globally important populations of seabirds and many endemic plants and animals, making them of considerable conservation importance^1–3^. The biotas of these islands and the ecosystems they constitute are nonetheless under considerable threat, in particular from climate change, biological invasions and their interactions^4–6^. Local population impacts and community re-arrangements attributable to these drivers have already been recorded from many of the islands^7–14^. Much is therefore being done to understand the likelihood of ongoing impacts and the ways in which they might be mitigated^15–17^.

One approach being used to determine impacts of climate change and invasion is to estimate the ways in which species abundances and distributions might be underpinned by climatic variation^18–23^. These studies have typically relied on either very coarse-resolution or very spatially-restricted climate data^19,22^, or some estimate of climate variation from elevation^18,21^. Those islands that have resident human populations or a research station typically only collect meteorological data from a single locality^24^.

Interpolated climate surfaces, such as the widely-used WorldClim2 dataset^25^, smooth between available weather stations for global land areas, using latitude, longitude and elevation^25,26^. The outcomes of models based on these interpolated and/or downscaled climatology datasets can be problematic where ground observations are sparsely available, because these methods can mask temporal and fine-scale climate variation and can potentially create false confidence in model outcomes^27–29^. Furthermore, as a result of the scarcity of ground observations, the error between interpolated climatology model predictions and observed climate increases in remote areas^26^. Areas identified with notably high prediction errors in the latest WorldClim2 dataset include oceanic islands, Greenland and Antarctica^26^. Estimations of climate variation based on simple elevational assumptions are likely to be even more prone to bias^28^.

In addition to their poor coverage by meteorological stations, the SOIs are also characterized by steep elevational gradients and strong prevailing winds which result in small-scale climate variation, including distinct windward and leeward thermal environments^24,30,31^. As climatology interpolation errors are often more pronounced across topographically complex and steep areas^26,29^, modelling the climates of these remote islands is particularly challenging. Nonetheless, given the importance of SOI biotas and ecosystems^1^, their anticipated vulnerability to environmental change^10,32^, and much investment in their management^16^, there is considerable value in providing high-quality environmental data. In these remote areas, the limitations of interpolated climatology datasets can be overcome by using remote-sensed data. For the SOI systems, which are typically not water limited^31,33,34^, temperature is especially important^35^ and frequently identified as a key factor influencing species abundances and distributions^10,19,22^. Moreover, because only a few of the most northerly islands have trees or shrubs^36^, surface temperature measurements are a useful approximation of local conditions.

Land surface temperatures can be observed remotely via satellite-mounted spectroradiometers that measure the amount of radiation reflected by the Earth’s surface^37^. Unlike interpolated climatology models, remote-sensed data are spatially and temporally continuous and more accurately describe climates in topographically complex and remote areas^28^. Remote-sensing methods are, however, restricted because their sensors require a clear sightline to accurately measure surface conditions^38^. Data obtained on days with heavy cloud or aerosol cover are, therefore, unusable. Datasets with missing values from cloud-cover can be analyzed in two ways, either choosing a statistical method that is robust to missing data, or predicting the missing values. Where missing values are non-randomly distributed, a prediction method is preferable^38^.

**Figure 1:**
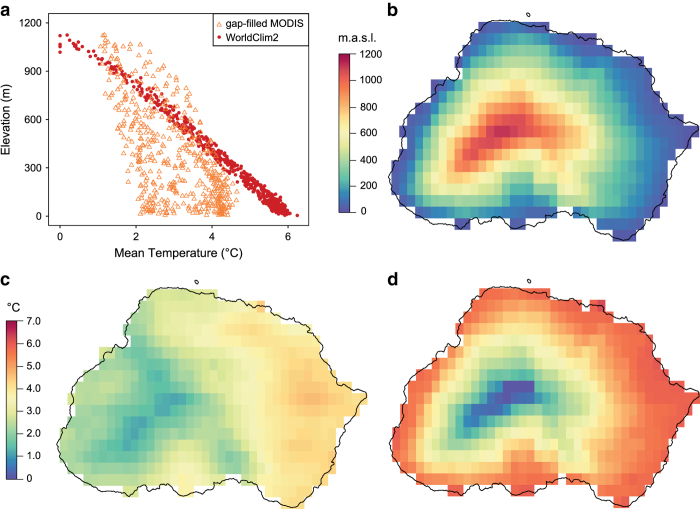
Collinearity of gap-filled MODIS land surface temperature data (this dataset) and WorldClim2 data with elevation on Marion Island. (**a**) Bivariate plot comparison of the collinearity with elevation of the gap-filled MODIS land surface temperature data (this dataset, orange open triangles) and WorldClim2 data (red filled dots). (**b**) Elevation. (**c**) Mean temperatures of the gap-filled MODIS land surface temperature data (this dataset). (**d**) Mean temperatures of the WorldClim2 data. Marion Island (−46.908 °S, 37.7424 °E) used as an example.

To reduce the number of missing remote-sensed land surface temperature (LST) observations, we applied a modified spatio-temporal gap-filling method^38^ to a monthly time-series (2001–2015) of high-resolution (1 km) LST observations from 20 SOIs. We validated results using standard gap-fill validation scenarios and fine-scale microclimate data from Marion Island. Our gap-filled temperature observations described the thermal heterogeneity of the region better than existing climatology datasets (e.g. Figure 1), especially for sub-Antarctic islands with steep elevational gradients and strong prevailing winds. Thus, we provide a regionally-specific, fine-scale temperature dataset, along with uncertainty measures and R code, for ecologists and conservation managers to better model species and ecosystem responses to climate change, and to develop strategies to manage and/or mitigate responses. These data also have value for understanding island climatology, geomorphological processes (such as diurnal soil frost and soil sorting^39,40^) and the evolutionary history of the biota^30,41–45^.

## Methods

### Remote-sensed MODIS land surface temperature data

High-resolution (~1 km) remote-sensed land surface temperature (LST) data were extracted from the Moderate Resolution Imaging Spectroradiometer (MODIS) Land Surface Temperature and Emissivity dataset (MOD11A2 Terra^37^). MODIS is a key contributor to the NASA Earth Observing System (EOS), which provides long-term global data on the state of Earth’s atmosphere, biosphere, land surface and oceans (see https://eospso.nasa.gov/). The MODIS sensor is mounted on the Terra satellite and has been observing every point on Earth once every 1 to 2 days in a near-polar, sun-synchronous orbit (altitude: 705 km; inclination: 98.1°), since its launch in December 1999 (ref. 37). MODIS is a multi-purpose, multi-spectral (36 bands), cross-track scanning instrument, that continuously observes several key atmospheric and surface variables, including aerosol properties, cloud cover, water vapor profiles, sea surface temperature, ocean color, surface albedo, fire intensities, snow and vegetation cover^37^. One of MODIS’s data products, the global LST and Emissivity 8-day dataset (MOD11A2) comprises 8-day average, clear-sky, day and night near-surface temperature observations (°K), stored on a 1 km Sinusoidal grid. This data product has been validated using ground-observations and other validation methods (e.g. an alternative radiance-based method) by MODIS’ land team^46,47^.

**Figure 2:**
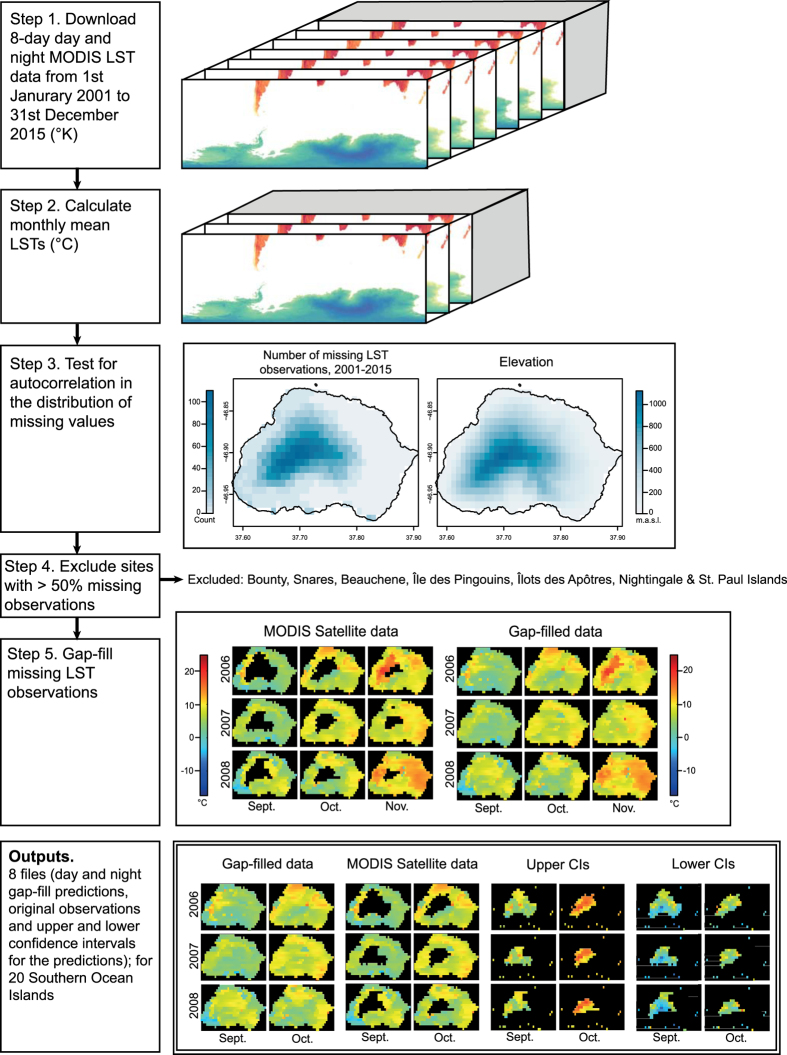
Gap-filling processing steps. Computational steps followed to develop the gap-filled, remote-sensed land surface temperature (LST) data outputs for the Southern Ocean Islands from January 2001 to December 2015. Marion Island (−46.908 °S, 37.7424 °E) used as an example.

MODIS Terra (MOD11A2 (ref. 37)) data were downloaded using the ‘MODIS’ R package (ver. 1.1.0 (ref. 48)), which uses the Geospatial Data Abstraction Library (GDAL; http://www.gdal.org/) to open, reproject and convert spatial data. All available daytime and nighttime land surface temperature data from the study region (>37 °S) and time period (January 2001 – December 2015) were downloaded and converted from the 1 km resolution MODIS Sinusoidal projection (SR-ORG:6842) to the WGS84 geographic coordinated system (EPSG:4326) at 0.0083° resolution using bilinear interpolation (Fig. 2, step 1). These MODIS datasets were then clipped to the spatial extent of each Southern Ocean Island (SOI) using high-resolution spatial shapefiles from the DIVA-GIS spatial data repository (http://www.diva-gis.org/data). Values were scaled using the MODIS conversion factor (0.02) to convert them to degrees Kelvin and then converted to degrees Celsius.

Though MODIS’s data products are run through validation procedures pre-publication^46,47^, occasional LST anomalies have been observed in polar regions due to the spectral similarities between cloud and snow cover in the visual bands^49–51^. In these instances, MODIS fails to distinguish between cloud and land surfaces (i.e. cloud contamination), and thus records erroneously extreme temperature observations^49,51^. For example, on the Antarctic Peninsula, several 8-day average temperatures between 2001 and 2015 were below −80 °C, with an absolute minimum 8-day average temperature of −124.01 °C. For this reason, in addition to the absence of high-resolution elevation models, Antarctica and the maritime Antarctic islands were not included in this dataset. Several of the SOIs are heavily glaciated (e.g. South Georgia, Heard) and may, therefore, be subject to these extreme LST anomalies caused by cloud contamination. Observations outside 99.99% quantiles in the 8-day MODIS observations, per island, were therefore excluded. The remaining data were averaged to produce monthly averages on a per island basis from 2001 to 2015 (Fig. 2, step 2). Hereafter, ‘MODIS’ refers to the monthly average LST data, derived from the global 8-day LST and Emissivity dataset (MOD11A2).

**Figure 3:**
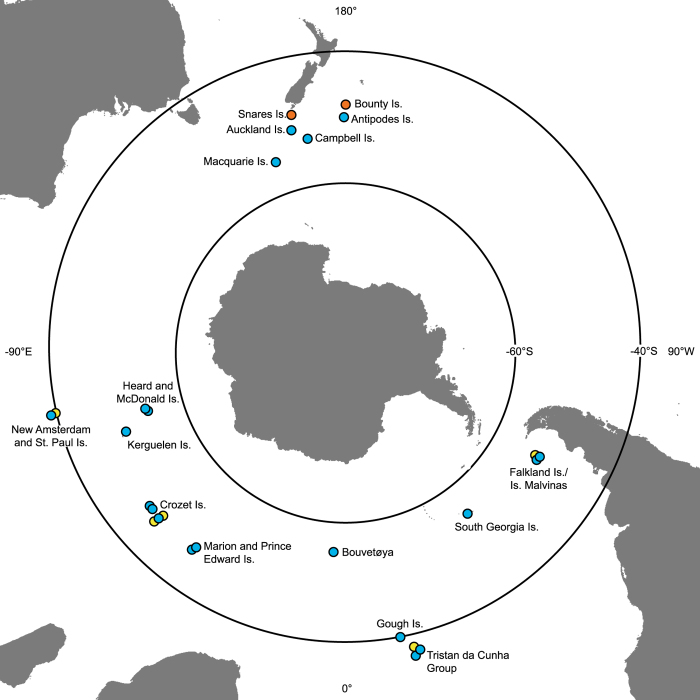
Map of the data availability of gap-filled MODIS monthly land surface temperature (LST) datasets for the Southern Ocean Islands from January 2001 to December 2015. Twenty islands have gap-filled data available (blue). Five islands were missing more than 50% of LST observations during the study period (yellow), while two islands had no remote-sensed LST observations (orange). For these islands (orange/yellow), gap-filled LST datasets are not available.

Here, we include the sub-Antarctic islands, cool-temperate Southern Ocean Islands (e.g. Tristan da Cunha group) and Falkland Islands/Islas Malvinas, but exclude maritime Antarctic islands due to data deficiency in high-resolution elevation models and the aforementioned cloud contamination issues (Fig. 3). Large amounts of MODIS data were available for 20 of the Southern Ocean Islands (see Table 1). For these islands, missing monthly mean LST observations were filled using the described gap-fill algorithm. The predicted mean values, along with 95% confidence intervals and the original MODIS data, are published here (Fig. 2 and Data Citation 1). For two small island groups, the Bounty Islands and Snares, no terrestrial MODIS observations were available in the MOD11A2 dataset for the study time period, 2001–2015. For a further five islands (Beauchene, Île des Pingouins, Îlots des Apôtres, Nightingale and St. Paul), more than 50% of the total monthly observations (cells) were missing and these islands were not gap-filled (Fig. 3).

### Non-random distribution of missing observations

Gap-filling predicts missing LST observations that occur due to cloud-contamination in remote-sensed data^38^. Missing observations can be problematic in many analyses, especially when they are spatially or temporally clustered, or skewed (e.g. towards colder values). For example, as missing observations (NAs) occur more frequently at high-elevation sites on Marion Island (Fig. 2, step 3), climatological summary statistics (e.g. mean annual temperature, minimum monthly temperature) calculated from available data and ignoring NAs would be skewed towards warmer values. To determine if missing observations in the SOIs data are non-randomly distributed and, therefore, suitable for the application of a gap-filling method, we undertook two preliminary analyses of the un-filled mean monthly MODIS observations.

First, Global Moran’s *I* tests were used to explicitly test for spatial autocorrelation in the frequency of missing monthly observations per spatial cell, over 15 years, using the ‘spdep’ package in R (ver. 0.6–13 (ref. 52)). Across the time series, the number of missing values (0 to 180) per cell in each island was calculated, where zero represented a site with complete data, and 180 a site with no observations at any time between 2001 and 2015 (e.g. Fig. 2, step 3). Second, a binomial generalized linear model (GLM, with a ‘logit’ link-function) was used to explore the relationship between the presence and absence of missing observations (where NA=1; data=0), and spatio-temporal factors (photoperiod (day/night), season, elevation). The daytime and nighttime MODIS observations, per spatial cell, across 24 islands (i.e. including Beauchene, Nightingale, Île des Pingouins and Îlots des Apôtres, that were later removed) were included in this analysis (n=16,440,660).

The preliminary analyses of the distribution of missing MODIS observations found significant spatial autocorrelation for 85.7% of the Southern Ocean Islands (Supplementary Table S1, Supplementary File 1). The occurrence of missing observations was significantly correlated with elevation, photoperiod and season (Supplementary Table S2, Supplementary File 1). Missing data were more likely to occur at higher elevations, during the daytime and spring months (Supplementary File 1). These preliminary results demonstrate the prevalence of both spatial and temporal clustering in the distribution of missing observations in the MODIS data, which may be problematic in subsequent analyses and models. It was, therefore, appropriate to apply a gap-filling method to these data.

### Gap-filling method

A gap-filling algorithm was used to interpolate missing LST values in the MODIS datasets (‘gapfill’ package, ver. 0.9.5–2 (ref. 38); Fig. 2, step 5). The gap-filling method applies a linear quantile regression to predict the value of missing observations, along with upper and lower 95% confidence interval estimates, based on the values of neighboring spatial cells and LST observations from neighboring months and years^38^. The number of neighboring cells used in this analysis is defined by the ‘gapfill’ ‘Subset’ function (‘gapfill’ package^38^). The gap-fill search strategy was defined to search across two spatial and two temporal dimensions, with sampling limited to five cells in every spatial (x, y) direction (i.e. an 11×11 grid centered on the target cell), and to include time points from the previous, same and next month (t_1_), and the previous and next two-year period (t_2_). To prevent the algorithm extrapolating missing data, clip ranges, which constrain the uppermost and lowermost values that the algorithm can predict, were set at the maximum and minimum observed LSTs for each island. This clip range applies only to the mean gap-fill values, therefore, confidence intervals can exceed the clip range.

The default functions of the ‘gapfill’ package were modified to incorporate digital elevation models (DEMs) into the quantile regression ‘Predict’ function (see code in Supplementary File 2). This modification aimed to improve the accuracy of the gap-fill predictions by including elevation as a model covariate, along with spatially and temporally neighboring LST observations, to account for the topographic heterogeneity of the study region. High-resolution (30 m) DEMs were sourced from the Shuttle Radar Topography Mission (SRTM^53^) and resampled using bilinear interpolation to the same spatial resolution (~1 km) and extent as the MODIS data.

For each SOI, the proportion of missing LST observations was calculated. The gap-fill algorithm was not applied to islands that had more than 50% missing observations (cells) across the 15-year time period in the monthly MODIS data (Beauchene, Île des Pingouins, Îlots des Apôtres, Nightingale and St. Paul) and for islands for which MODIS LST data were unavailable (Bounty and Snares; Fig. 2, step 4 and Fig. 3). The gap-fill analysis failed to predict any of the missing LST observations for the McDonald islands because the missing values occurred across all cells within a given month, and thus, within the defined gap-fill subset, there were no observations for neighboring spatial cells.

Summary statistics, on a per island basis, were calculated, including mean LST, LST range from the mean spatial layer across the time period (a proxy for thermal niche diversity), absolute observed maximum LST, absolute observed minimum LST and the percentage of data available in the original MODIS observations (results presented in Table 1).

### Dataset comparison

The gap-filled MODIS LST data presented here differs from other commonly-used climate datasets, including WorldClim2^25^ and NASA’s Earth Exchange Global Daily Downscaled Projections (NEX-GDDP) dataset^54^, with regard to spatial and temporal resolution. NEX-GDDP data have a coarser spatial resolution than MODIS and WorldClim2 data (NEX-GDDP: 0.25°; MODIS/WorldClim2: 0.0083°), making NEX-GDDP of limited use in exploring fine-scale intra-island thermal variation. WorldClim2 temperature data are long-term average monthly temperatures, interpolated between weather stations using covariates such as latitude and elevation^25^. In remote locations, including oceanic islands, WorldClim2 data have high prediction errors arising from the low density of meteorological stations^25^. The inclusion of remote-sensed MODIS LSTs as covariates in the WorldClim2 interpolation was intended to improve estimates for remote areas, however, such improvements were negligible and high prediction errors for remote locations remain^25^. Furthermore, in the absence of multiple weather stations, the WorldClim2 estimates for the Southern Ocean Islands are highly co-linear with elevation (Fig. 1a). Consequently, gap-filled MODIS data describes thermal heterogeneity, including the distinct windward and leeward thermal environments that are characteristic of the sub-Antarctic islands with steep elevational gradients and strong prevailing winds, better than WorldClim2 and NEX-GDDP datasets.

### Code availability

A complete worked example of the methods, from data download to the gap-filled data records, including the modified version of the ‘gapfill’ ‘Predict’ function code used to incorporate digital elevation models into the gap-fill quantile regressions (‘gapfill’, ver. 0.9.5–2 (ref. 38)), is supplied in the Supplementary Information (Supplementary File 2). The code was written in R statistical software (ver. 3.3.3 (ref. 55)). Where applicable, Marion Island (−46.908 °S, 37.7424 °E; Fig. 3) is used as an example.

## Data Records

The data records contain validated, gap-filled, mean monthly, high resolution (0.0083°) land surface temperature (LST) data, in °C, for 20 Southern Ocean Islands for the study time period, January 2001 to December 2015 (see list in Table 1). For each island, LSTs are divided into daytime and nighttime observations (photoperiods). Four types of data are available: gap-fill mean predictions (abbreviated as ‘mean’), upper confidence intervals (abbreviated as ‘upperCI’), lower confidence intervals (abbreviated as ‘lowerCI’) and observations (abbreviated as ‘obs’). The ‘mean’ data records comprise the original MODIS observations and gap-filled mean LST predictions for missing observations (i.e. the most complete records). The upper and lower 95% confidence interval data records provide confidence intervals for the gap-fill estimates (NA for original MODIS observations). The ‘observations’ data type contain the un-filled mean monthly MODIS LST observations (i.e. pre-gapfill).

Each data record is available in both a netCDF (.nc) and native raster package format (.grd; R ‘raster’ package, ver. 2.5–8 (ref. 56)), with 180 data layers per file, ordered sequentially (band name format: YYYYMM). All files have a geographic coordinate reference system (WGS 84 EPSG:4326), with coordinates expressed in decimal degrees. The files are freely available at *Figshare* (Data Citation 1), compressed per island using the zip file format.

### Naming convention:

<island name>_<photoperiod>_1km_mon_<data type>.grd

e.g.

- Marion_Day_1km_mon_mean.nc- contains mean monthly predictions and observations for daytime LSTs on Marion Island (~1 km resolution).
- Kerguelen_Night_1km_mon_lowerCI.grd- contains lower 95% confidence intervals for mean nighttime predictions for the Kerguelen Islands.

Additionally, the mean monthly, day and night soil temperatures, in °C, from nine sites on Marion Island from May 2002 to May 2013, used to ground-validate the accuracy of the gap-filled remote-sensed MODIS data (see Technical Validation; site details presented in Supplementary Table S3, Supplementary File 1), are also freely available in Data Citation 1. These data are provided in a comma-separated values (.csv) file.

## Technical Validation

The gap-fill predictions were validated in three ways. First, by applying two sets of validation scenarios to quantify prediction error, where observations were randomly deleted in either points or spatial clusters to mimic observed patterns of missing observations. The gap-filled MODIS data were also ground-validated with fine-scale soil temperature data from Marion Island. Additionally, analyses were also applied to identify non-random spatial patterns in the distribution of gap-fill prediction errors.

### Gap-fill validation scenarios- quantifying prediction accuracy under different support scenarios

#### Random knockout scenarios

To evaluate the accuracy of the gap-fill predictions, the validation scenarios developed by Gerber and colleagues^38^ were applied to the original, un-filled monthly MODIS data for the Southern Ocean Islands. Six scenarios were run, where 5, 10, 20, 30, 40 and 50% of the original observations (cells) per island were randomly removed. The remaining data were gap-filled (see Methods) and the gap-fill predictions were compared to the removed observations. This validation method was only applied to islands that had fewer than 10% missing observations in the original data (n=13; East Falklands, West Falklands, Tristan da Cunha, Gough, Prince Edward, Marion, Île aux Cochons, Île de l’Est, Île de la Possession, New Amsterdam, Macquarie, Campbell and Auckland Islands). The accuracy and precision of the gap-fill predictions were quantified using several error statistics, including the root mean squared error (RMSE), mean error (the average difference (in °C) between the observed and predicted values), absolute error range (maximum error - minimum error), standard deviation of the error distribution, and the number of times where the gap-fill method failed to predict a missing value.

#### Clustered knockout scenarios

A second set of gap-fill validation scenarios was applied to the un-filled MODIS data of the same 13 islands, where the original LST observations were deleted in random spatial clusters (3×3 grids), instead of random points, to mimic observed patterns of spatial autocorrelation in the distribution of missing observations (see Supplementary File 1). In these scenarios, approximately 5, 10, 20, 30, 40 and 50% of the original observations were removed using a random seed value to select center cells, from which the adjoining cells in every spatial direction and the center cell were deleted. The remaining data were gap-filled (see Methods).

In these spatially-clustered validations, the relationship between gap-fill prediction errors (the absolute difference between the observed and predicted LST values) and support was quantified. Support for a given prediction (cell) is the number of spatially and temporally neighboring cells with observed data used in the gap-fill analysis to estimate the missing value. Gap-fill predictions for cells with few observed values within the defined spatio-temporal search strategy (i.e. those with low support) are expected to be less accurate estimates of observed land surface temperatures than cells surrounded by high levels of support. We therefore expect gap-fill prediction error to increase with decreasing support.

Absolute error is left censored at zero and consistently demonstrated unequal variance with support. Given unequal variance, the relationships between error and support for each validation scenario were analyzed using a linear quantile regression, for the median (0.5) quantile, using the ‘quantreg’ package in R (ver. 5.33; Koenker 2017)^57^. Quantile regressions make no assumptions about the distribution of the errors and are, therefore, more robust to unequal variance and outliers than linear regressions^58^. Here, quantile regressions were used to determine whether there are, on average, significant relationships between prediction error and support across validation scenarios where increasingly large amounts of data were deleted in spatial clusters. Goodness-of-fit criterion (pseudo-*r*^2^ values) were calculated for each quantile regression as the weighted sum of the absolute residuals^59^.

#### Non-random distribution of gap-fill errors

To identify non-random distributions in the occurrence of gap-fill prediction errors, two analyses were applied to the prediction errors of the 10% random point knockout validation scenarios. These analyses mirrored the autocorrelation tests conducted on MODIS gap data themselves (see Non-random distribution of missing observations). As an inherent property of filling data gaps based on spatio-temporal neighbors, autocorrelation of gap-fill errors is expected. Identifying the form of this autocorrelation, in addition to the confidence interval estimates provided in Data Citation 1, is a valuable exercise for evaluating model uncertainty. The 10% random validations were analyzed because they most closely resemble the average percentage of missing data in the twenty SOIs in this dataset (9.85%). For these analyses, the frequency of gap-fill errors (the absolute difference between observed and predicted LSTs) greater than 1 °C was calculated per spatial cell across each island. A value of zero, therefore, represented a site where gap-fill predictions were always within a degree of the observed LST, and a non-zero count represented the number of times the gap-fill predictions for that site were more than 1 °C from observed temperatures across the 15-year time period. First, Global Moran’s *I* tests were used to test for spatial autocorrelation in the frequency of gap-fill errors greater than 1 °C per spatial cell, using the ‘spdep’ package in R (ver. 0.6–13 (ref. 52)). This test was applied to determine whether prediction errors greater than 1 °C were spatially-clustered (Supplementary Table S4, Supplementary File 1). Next, a negative binomial generalized linear model (GLM) was used to explore relationships between the frequency of gap-fill prediction errors greater than 1 °C per spatial cell and spatio-temporal factors (photoperiod, elevation). The GLM was applied to prediction errors from the 10% random knockout scenarios across 13 islands, where the response variable was a count of the number of months across the 15-year time period where the gap-fill prediction error was greater than 1 °C from the observed LST (n=47,088).

### Ground truthing- comparing gap-fill predictions and observed remote-sensed temperatures to long-term, fine-scale ground observations on Marion Island

To ground truth the gap-filled LST predictions, the difference between gap-fill predictions and soil microclimate temperatures was compared to the difference between the un-filled MODIS observations and soil microclimate temperatures. The microclimate data comprises soil temperatures recorded hourly from May 2002 to May 2013 at nine sites along an elevational gradient on Marion Island (see Supplementary Table S3, Supplementary File 1, for site details). Data-loggers (Thermochron iButton, DS1921G & DS1922L-F5, Maxim Integrated, San Jose, USA; accuracy: 0.5–1.0 °C) were placed (approximately 1–2 cm below the surface) along an upslope transect from 0 m to 800 m, at roughly 100 m elevation intervals. Conspicuously erroneous records (e.g. where the soil temperature spiked +10 °C in one hour before the iButton failed) were removed manually. The soil microclimate temperatures were divided into daytime and nighttime records by calculating the time of sunrise and sunset each day over the time period, per site, using the ‘insol’ package in R (ver. 1.1.1 (ref. 60)). Average monthly soil temperatures (Mean monthly soil temperatures Marion Island, Data Citation 1) were then calculated as the mean monthly daytime and nighttime soil temperature of each site from May 2002 to May 2013.

To determine whether there is a difference in how closely gap-filled LST predictions and un-filled LST observations reflect mean monthly soil temperatures on Marion Island, two statistical approaches were applied. First, Pearson’s correlation coefficients (*r*) for the relationships between daytime and nighttime LST values (gap-filled vs. un-filled) and soil temperatures were calculated. Strong correlations between land surface and soil temperatures are unlikely because soil, vegetation and snow insulate soil microclimates, making them less variable than surface conditions^61^. If, however, gap-fill predictions are much weaker correlates of soil temperatures than the original MODIS observations, then the gap-filled estimates would be unreliable indicators of ground conditions. Second, Mann-Whitney *U* tests were used to determine whether there are significant differences in the absolute errors of the daytime and nighttime gap-filled versus un-filled LST values from soil microclimate temperatures. In this analysis, error is the absolute difference between land surface and soil temperatures. Effect sizes estimates (*r*) were calculated by dividing the *z*-value by the square root of the total sample size (*n*).

Fifty percent of the LST observations were randomly deleted, prior to gap-fill and analysis because very few (<3%) of the original MODIS LST observations were missing at the microclimate sites during the study period. This knockout was applied to ensure a more equal sample size between the gap-filled and un-filled data. Additionally, because snow buffers soil temperatures^61^, the relationship between soil and near-surface temperatures weakens considerably in sub-zero conditions. Months with average soil temperatures below 0 °C (fewer than 5% of observations) were therefore excluded from the correlations and Mann-Whitney *U* tests. The root mean squared errors (RMSE) of the daytime and nighttime gap-fill predictions and un-filled MODIS observations from soil microclimate temperatures were calculated to quantity the relative differences between land surface and soil temperatures.

## Validation Results

### Random knockout results

In the first set of validation scenarios, the gap-fill algorithm predicted all randomly deleted values in every scenario (Table 2 (available online only)). The root mean squared error (RMSE) and mean error did not increase substantially across validation scenarios, where increasingly large amounts of observed data were artificially removed (Table 2 (available online only)). The mean error was greater than 1 °C in only three cases (Tristan da Cunha day and night LSTs, New Amsterdam night LST), where the predicted temperatures were, on average, warmer than the observed temperatures (Table 2 (available online only)). Absolute error ranges, the difference between the maximum and minimum errors, increased marginally across validation scenarios (Table 2 (available online only)). Likewise, the standard deviations of the errors, a measure of gap-fill precision, were highly consistent across validation scenarios (Table 2 (available online only)). Overall, the consistency in prediction errors across validation scenarios suggests that the gap-fill predictions are accurate indications of mean monthly land surface temperatures, even when relatively large amounts of observations are missing (Table 2 (available online only)).

### Clustered knockout results

In the second set of validations, where observed LSTs were deleted in spatial clusters, relationships between gap-fill prediction error and support (i.e. the number of spatially and temporally neighboring cells with observed LSTs used to predict missing values) varied across validation scenarios and islands (Table 3 (available online only)). In most scenarios (71.1%), there was either no relationship or a weak positive relationship between prediction error and support, contrary to the expectation that prediction error should be smaller for cells surrounded by high levels of support (many neighboring observations) (Table 3 (available online only)).

Weak negative relationships between prediction error and support occurred in fewer than 29% of the validation scenarios at the median (0.5) quantile (Table 3 (available online only)). In these scenarios, there was a significant, though small, reduction in gap-fill prediction error with increasing support. The number of models with a significant negative relationship between prediction error and support did not increase substantially across validation scenarios, where increasing large amounts of observed LST data were deleted in spatial clusters (Table 3 (available online only)). Four islands (Auckland, Macquarie and East and West Falklands), of the thirteen tested, showed consistently declining trends between error and support (Table 3 (available online only)).

The slopes of all quantile regressions were shallow (<0.01), indicating that although the trends may be significant, the average increase or decrease in prediction errors across the range of support values was small (Table 3 (available online only) and Supplementary Figure S1, Supplementary File 1). For example, in the scenario with the strongest negative relationship between prediction error and support, the daytime Macquarie Island validation, where approximately 50% of the observed LSTs were deleted, the average increase in gap-fill prediction error from a high-support cell (1000 spatially and temporally neighboring cells with data) to a low-support cell (100 neighboring cells with data) was 0.54 °C. Likewise, the goodness-of-fit values (pseudo *r*^2^) for all models were small (<0.15; Table 3 (available online only)), indicating that prediction errors are highly variable across support values. Overall, the absence of consistent, strong negative relationships between gap-fill prediction error and support across validation scenarios and islands indicates that the mean gap-fill predictions are robust estimates of LSTs, even where large amounts of neighboring observations are missing.

### Error distribution results

Of the 47,088 gaps filled in the 10% random knockout scenarios, 71.93% had an absolute gap-fill error of less than or equal to 1 °C. The frequency of gap-fill prediction errors greater than 1 °C was significantly spatially auto-correlated in the 10% random validation scenarios (Supplementary Table S4,Supplementary File 1). These errors greater than 1 °C occurred more frequently at higher elevations and during the daytime (Supplementary Table S5, Supplementary File 1). Nighttime prediction errors greater than 1 °C tended to be more spatially-clustered (higher Moran’s I statistics, Supplementary Table S4, Supplementary File 1), yet occurred less frequently, than daytime prediction errors. The observed increase in error frequency with elevation and photoperiod was, however, relatively small (<0.01 °C m^−1^, 0.77 °C day/night on average, respectively; Supplementary Table S5, Supplementary File 1), as were mean and standard deviation error values (Table 2 (available online only)). These findings indicate greater uncertainty in gap-fill predictions for missing LSTs at high elevation sites and during the daytime in the SOIs dataset (Data Citation 1).

### Ground validation results

Daytime gap-fill predictions were marginally weaker correlates of Marion Island soil microclimate temperatures than un-filled MODIS LST observations (day gap-fill *r*: 0.60, n=567; day observations *r*:0.65, n=549). Conversely, nighttime gap-fill predictions were more strongly correlated with soil temperatures than nighttime LST observations (night gap-fill *r*: 0.70, n=526; night observations *r*: 0.61, n=525).

There was no significant difference between the median absolute errors of the daytime gap-filled and un-filled LST values from soil temperatures on Marion Island (*U*= 154270, *p*=0.799, *r*=<0.01; day gap-fill: RMSE=4.87, median=3.27, IQR=4.10, n=567; day observations: RMSE=4.93, median=3.26, IQR=3.95, n=550). The nighttime gap-fill predictions had a significantly lower median error value from soil temperatures than nighttime LST observations (*U*=104520, *p* <0.001, *r*=-0.18; night gap-fill: RMSE=3.49, median=3.18, IQR=1.79, n=526; night observations: RMSE=4.29, median=3.98, IQR=2.34, n=525). Nighttime gap-fill predictions are, therefore, more similar to soil microclimate temperatures than un-filled MODIS LST observations. This likely arises because nighttime soil temperatures tend to be warmer and less variable than surface temperatures, due to the insulating effects of soil^61^. Gap-fill predictions are derived from a quantile regression of neighboring data and will, therefore, reflect central tendency rather than more extreme LST values (e.g. unseasonably cold nights). Thus, nighttime gap-fill predictions may be more similar to soil microclimate conditions than more variable surface temperatures.

## Usage Notes

The data records are available in netCDF and native raster package data formats. These can be viewed in standard GIS software, including:

ArcGIS- https://www.arcgis.com

QGIS- http://www.qgis.org/

R- https://www.r-project.org/

To view netCDF data files in R, the ‘ncdf4’ (ver. 1.16 (ref. 62)) and ‘raster’ (ver. 2.5-8 (ref. 56)) packages may be required.

As a consequence of the LST anomalies caused by cloud contamination, known to affect MODIS observations in polar regions^49,50^ (see discussion in Methods), and the prevalence of missing observations at high elevations, some gap-fill estimates in the daytime data records for Kerguelen, Heard and South Georgia have extremely large confidence intervals (e.g. the maximum upper 95% confidence interval value on Heard Island was 92.78 °C). It may be appropriate to exclude some gap-fill estimates with extremely large confidence intervals from analyses of these islands. Likewise, gap-fill prediction errors greater than 1 °C occur more frequently at high elevation sites and during the daytime across the Southern Ocean Islands (see Technical Validation and Supplementary Table S5, Supplementary File 1). Gap-fill predictions are, therefore, likely to be less accurate estimates of LSTs under these conditions, however, the observed increase in error frequency across these spatio-temporal gradients was small.

## Additional information

**How to cite this article**: Leihy, R. I. *et al*. High resolution temperature data for ecological research and management on the Southern Ocean Islands. *Sci. Data* 5:180177 doi: 10.1038/sdata.2018.177 (2018).

**Publisher’s note**: Springer Nature remains neutral with regard to jurisdictional claims in published maps and institutional affiliations.

## Supplementary Material



Supplementary File 1

Supplementary Information

## Figures and Tables

**Table 1:** Summary statistics for the gap-filled, mean monthly remote-sensed land surface temperature (LST) datasets, derived from MODIS observations, for the Southern Ocean Islands, 2001–2015.

**Island**	**Biogeographic Region**	**Area (kms**^**2**^)	**Mean LST (°C)**		**LST Range (°C)**		**Absolute Max (°C)**	**Absolute Min (°C)**	**% Data Available**		**% Data Available** (post-gapfill)		**Notes**
			**Day**	**Night**	**Day**	**Night**			**Day**	**Night**	**Day**	**Night**	
Antipodes	Southern Pacific Ocean	22.9	8.26	2.23	2.54	1.71	18.17	−6.81	70.33	89.87	85.76	100	
Auckland	Southern Pacific Ocean	598.6	6.01	1.31	3.87	5.44	21.27	−12.67	97.36	99.82	100	100	
Beauchene*	Falkland Islands	2.1	*NA*	*NA*	*NA*	*NA*	11.29	−14.09	37.22	60.83	*NA*	*NA*	> 50% data missing
Bounty*	Southern Pacific Ocean	0.7	*NA*	*NA*	*NA*	*NA*	*NA*	*NA*	*NA*	*NA*	*NA*	*NA*	> 50% data missing
Bouvetøya	Southern Atlantic Ocean	77.6	−3.25	−6.34	2.97	3.81	5.87	−20.19	41.12	97.77	72.42	100	
Campbell	Southern Pacific Ocean	113	5.23	0.87	1.84	3.51	16.95	−12.27	97.08	99.71	100	100	
East Falkland	Falkland Islands	6614.3	9.41	0.68	4.39	5.33	27.74	−16.51	99.71	99.85	100	100	
Gough	Southern Atlantic Ocean	72.1	10.71	5.44	2.41	3.84	25.37	−7.47	92.92	98.71	100	100	
Heard	Southern Indian Ocean	359.3	−1.34	−6.16	8.49	7.43	17.33	−32.71	46.13	96.01	97.78	100	
Île aux Cochons	Southern Indian Ocean	70.1	6.81	−0.1	4.67	3.12	28.41	−9.45	96.82	99.96	100	100	
Île de l'Est	Southern Indian Ocean	138	6.26	−1.07	5.81	3.7	25.11	−18.27	95.2	99.96	100	100	
Île de la Possession	Southern Indian Ocean	156.5	6.56	−0.98	5.62	4.24	25.83	−18.27	94.39	99.98	100	100	
Île des Pingouins*	Southern Indian Ocean	3.8	*NA*	*NA*	*NA*	*NA*	12.83	−9.27	32.36	59.58	*NA*	*NA*	> 50% data missing
Îlots des Apôtres*	Southern Indian Ocean	2.9	*NA*	*NA*	*NA*	*NA*	12.71	−8.39	2.22	50	*NA*	*NA*	> 50% data missing
Inaccessible	Southern Atlantic Ocean	14.7	14.11	8.83	1.29	1.97	24.07	0.76	65.7	94.71	70.53	97.87	
Kerguelen	Southern Indian Ocean	7337.6	4.62	−3.2	8.11	10.71	24.73	−20.87	81.38	97.49	100	100	
Macquarie	Southern Pacific Ocean	134.2	3.85	−2.31	1.81	3.32	21.29	−14.61	93.02	99.65	100	100	
Marion	Southern Indian Ocean	296.9	6.86	−0.87	3.97	5.87	25.65	−17.53	90.48	99.69	100	100	
McDonald	Southern Indian Ocean	2.5	2.38	−2.99	0.41	0.08	14.65	−10.77	45.56	80.33	45.56	80.33	Gap−fill failed
New Amsterdam	Southern Indian Ocean	54.9	14.66	7.72	5.05	3.87	30.91	−3.57	96.41	99.84	100	100	
Nightingale*	Southern Atlantic Ocean	3.5	*NA*	*NA*	*NA*	*NA*	19.13	*NA*	*NA*	1.11	*NA*	*NA*	> 50% data missing
Prince Edward	Southern Indian Ocean	46.4	7.04	0.04	2.45	2.2	21.03	−8.13	96.1	99.93	99.44	100	
Snares*	Southern Pacific Ocean	5.4	*NA*	*NA*	*NA*	*NA*	*NA*	*NA*	*NA*	*NA*	*NA*	*NA*	> 50% data missing
South Georgia	Southern Atlantic Ocean	3752.3	−2.47	−9.18	13.83	15.68	21.67	−36.77	56.46	99.64	100	100	
St Paul*	Southern Indian Ocean	9.2	*NA*	*NA*	*NA*	*NA*	*NA*	0.61	*NA*	1.06	*NA*	*NA*	> 50% data missing
Tristan da Cunha	Southern Atlantic Ocean	97.8	12.97	6.54	4.83	6.73	32.47	−3.19	98.16	99.24	100	100	
West Falkland	Falkland Islands	4644	9.32	1.07	5.05	5.11	28.95	−15.43	99.61	99.91	100	100	
*Islands with more than 50% missing observations in the original MODIS datasets were not gap-filled.													

**Table 2:** Gap-fill validation outcomes for six validation scenarios, where 5, 10, 20, 30, 40 and 50% of observed (un-filled) remote-sensed MODIS land surface temperature (LST) data were randomly removed and subsequently gap-filled.

**Site**	**Validation Scenario**	**Day LST Dataset**				**Night LST Dataset**			
		RMSE	Mean Error (°C)	Error Range (°C)	Error SD	RMSE	Mean Error (°C)	Error Range (°C)	Error SD
Auckland	5%	1.47	−0.24	19.68	1.45	1.04	−0.56	11.20	0.88
	10%	1.44	−0.23	20.55	1.42	1.01	−0.56	13.48	0.84
	20%	1.50	−0.24	23.76	1.48	1.05	−0.57	14.85	0.88
	30%	1.52	−0.25	26.33	1.50	1.06	−0.58	15.59	0.89
	40%	1.53	−0.25	26.82	1.51	1.07	−0.58	18.69	0.90
	50%	1.54	−0.28	25.90	1.51	1.09	−0.61	18.96	0.91
Campbell	5%	1.28	−0.37	10.33	1.23	1.07	−0.55	11.28	0.93
	10%	1.29	−0.36	14.51	1.24	1.01	−0.53	12.98	0.85
	20%	1.34	−0.36	18.55	1.29	1.06	−0.54	12.70	0.91
	30%	1.36	−0.39	18.94	1.31	1.05	−0.53	12.34	0.90
	40%	1.36	−0.39	16.96	1.30	1.08	−0.57	16.30	0.92
	50%	1.38	−0.38	20.48	1.33	1.09	−0.57	16.94	0.93
East Falkland	5%	1.38	0.06	24.51	1.38	0.79	−0.21	17.94	0.76
	10%	1.30	0.07	23.25	1.30	0.75	−0.21	19.09	0.73
	20%	1.39	0.06	27.74	1.39	0.79	−0.21	20.89	0.76
	30%	1.40	0.06	28.53	1.39	0.80	−0.22	21.41	0.77
	40%	1.40	0.05	31.18	1.40	0.81	−0.22	23.87	0.78
	50%	1.42	0.04	29.14	1.42	0.82	−0.22	21.78	0.79
Gough	5%	1.90	<0.01	24.28	1.90	1.56	−1.20	8.06	1.01
	10%	1.89	−0.15	18.67	1.88	1.54	−1.14	10.05	1.04
	20%	1.95	−0.17	25.34	1.94	1.61	−1.18	16.39	1.10
	30%	1.97	−0.12	26.26	1.97	1.62	−1.19	14.28	1.10
	40%	1.97	−0.17	26.15	1.96	1.63	−1.18	15.38	1.12
	50%	2.00	−0.16	26.39	2.00	1.63	−1.16	16.86	1.14
Île aux Cochons	5%	1.82	<0.01	21.81	1.82	1.11	−0.54	8.34	0.97
	10%	1.77	−0.01	18.41	1.77	1.05	−0.50	10.77	0.93
	20%	1.82	<0.01	24.44	1.82	1.08	−0.50	12.61	0.96
	30%	1.82	−0.04	25.88	1.82	1.10	−0.51	11.25	0.97
	40%	1.78	−0.07	24.00	1.78	1.10	−0.51	9.96	0.97
	50%	1.82	−0.11	25.25	1.82	1.13	−0.54	12.79	0.99
Île de l'Est	5%	1.91	−0.13	22.57	1.91	1.16	−0.36	13.54	1.10
	10%	1.84	−0.13	28.25	1.84	1.10	−0.40	12.51	1.03
	20%	1.91	−0.17	33.47	1.90	1.14	−0.39	12.39	1.07
	30%	1.92	−0.12	31.23	1.92	1.15	−0.42	12.90	1.07
	40%	1.96	−0.17	29.54	1.95	1.15	−0.41	17.81	1.07
	50%	2.00	−0.17	33.95	1.99	1.17	−0.40	18.20	1.10
Île de la Possession	5%	1.87	−0.28	20.06	1.85	1.20	−0.66	10.14	1.01
	10%	1.84	−0.36	28.57	1.81	1.18	−0.64	15.82	0.99
	20%	1.99	−0.36	32.06	1.96	1.21	−0.61	13.08	1.04
	30%	1.96	−0.43	31.06	1.91	1.24	−0.64	13.66	1.06
	40%	1.99	−0.39	32.28	1.96	1.26	−0.65	15.63	1.08
	50%	2.02	−0.45	32.77	1.96	1.27	−0.66	15.72	1.08
Macquarie	5%	1.47	−0.07	13.65	1.46	1.01	−0.27	12.04	0.97
	10%	1.50	−0.09	20.07	1.50	1.00	−0.32	15.38	0.95
	20%	1.58	−0.11	25.46	1.57	1.02	−0.28	15.14	0.98
	30%	1.53	−0.06	23.62	1.53	1.02	−0.30	14.55	0.98
	40%	1.60	−0.13	26.20	1.60	1.06	−0.31	15.24	1.01
	50%	1.62	−0.13	25.51	1.62	1.07	−0.32	18.25	1.02
Marion	5%	1.96	−0.50	28.68	1.89	1.58	−0.87	14.48	1.32
	10%	1.91	−0.52	30.65	1.84	1.61	−0.89	18.19	1.35
	20%	2.00	−0.52	34.78	1.94	1.64	−0.89	23.59	1.38
	30%	2.01	−0.52	35.46	1.94	1.64	−0.89	20.52	1.38
	40%	2.03	−0.54	32.90	1.95	1.65	−0.91	21.78	1.38
	50%	2.05	−0.53	35.11	1.98	1.66	−0.89	24.50	1.40
New Amsterdam	5%	1.96	−1.03	17.10	1.67	1.30	−1.04	7.35	0.78
	10%	1.88	−0.98	18.18	1.61	1.34	−1.08	9.19	0.79
	20%	1.89	−0.98	20.15	1.62	1.35	−1.10	9.31	0.78
	30%	1.89	−0.97	18.78	1.62	1.36	−1.12	7.88	0.77
	40%	1.92	−1.02	19.68	1.62	1.39	−1.13	10.06	0.81
	50%	1.95	−1.02	24.33	1.66	1.38	−1.13	8.86	0.80
Prince Edward	5%	1.45	−0.51	12.00	1.36	1.08	−0.55	9.03	0.93
	10%	1.53	−0.45	13.65	1.47	0.97	−0.56	7.98	0.80
	20%	1.59	−0.47	19.99	1.52	1.03	−0.58	8.27	0.85
	30%	1.58	−0.45	19.11	1.52	1.00	−0.56	9.07	0.83
	40%	1.58	−0.51	19.93	1.49	1.03	−0.57	9.77	0.86
	50%	1.63	−0.42	19.65	1.58	1.05	−0.58	9.85	0.87
Tristan	5%	2.15	−0.82	21.12	1.99	2.89	−2.58	9.38	1.31
	10%	2.03	−0.75	22.01	1.88	2.86	−2.57	10.79	1.26
	20%	2.12	−0.76	28.37	1.97	2.88	−2.56	12.76	1.31
	30%	2.09	−0.81	20.91	1.92	2.91	−2.61	12.86	1.29
	40%	2.14	−0.81	28.92	1.99	2.92	−2.60	14.09	1.33
	50%	2.15	−0.80	30.05	2.00	2.94	−2.61	14.99	1.36
West Falkland	5%	1.30	−0.17	27.69	1.29	0.79	−0.29	15.87	0.73
	10%	1.22	−0.17	24.66	1.20	0.75	−0.29	17.89	0.70
	20%	1.31	−0.17	26.13	1.30	0.79	−0.29	20.56	0.74
	30%	1.32	−0.18	31.08	1.31	0.80	−0.29	19.77	0.74
	40%	1.33	−0.19	30.42	1.32	0.81	−0.30	23.57	0.75
	50%	1.34	−0.19	27.84	1.33	0.82	−0.30	22.22	0.76
*islands with more than 50% missing observations in the original MODIS datasets were not gap-filled.									

**Table 3:** Gap-fill validation outcomes for six validation scenarios, where approximately 5, 10, 20, 30, 40 and 50% of observed (un-filled) remote-sensed MODIS land surface temperature (LST) data were deleted in random spatial clusters to mimic observed patterns of missing observations and subsequently gap-filled.

Site	Validation Scenario	Day LST Data								Night LST Data							
		Mean no. support	Min no. support	slope (SE)	Pseudo r^2^	*t*	resid. df	*P*	Trend	Mean no. support	Min no. support	slope (SE)	Pseudo r^2^	*t*	resid. df	*P*	Trend
Auckland	5%	1062.39	38	<0.01 (<0.01)	<0.01	−3.69	8137	<0.001	**significant decline**	1085.99	43	<0.01 (<0.01)	<0.01	−2.17	8498	0.030	**significant decline**
	10%	1019.33	23	<0.01 (<0.01)	<0.01	−1.77	15887	0.077	no relationship	1097.88	48	<0.01 (<0.01)	<0.01	−0.77	8425	0.443	no relationship
	20%	932.56	32	<0.01 (<0.01)	<0.01	−7.55	30889	<0.001	**significant decline**	945.38	39	<0.01 (<0.01)	<0.01	3.10	31694	0.002	significant increase
	30%	850.26	33	<0.01 (<0.01)	<0.01	−9.27	44564	<0.001	**significant decline**	861.02	22	<0.01 (<0.01)	<0.01	1.74	45739	0.082	no relationship
	40%	771.69	23	<0.01 (<0.01)	<0.01	−9.56	57089	<0.001	**significant decline**	682.10	26	<0.01 (<0.01)	<0.01	−0.28	75311	0.777	no relationship
	50%	705.25	24	<0.01 (<0.01)	<0.01	−10.85	68357	<0.001	**significant decline**	708.73	28	<0.01 (<0.01)	<0.01	1.65	70837	0.099	no relationship
Campbell	5%	853.90	142	<0.01 (<0.01)	<0.01	1.02	1558	0.308	no relationship	828.20	116	<0.01 (<0.01)	<0.01	−0.49	1551	0.626	no relationship
	10%	811.68	135	<0.01 (<0.01)	<0.01	−2.28	2980	0.022	**significant decline**	843.79	131	<0.01 (<0.01)	<0.01	−0.77	1549	0.439	no relationship
	20%	721.17	102	<0.01 (<0.01)	<0.01	0.24	5699	0.813	no relationship	735.23	97	<0.01 (<0.01)	<0.01	−1.07	5842	0.285	no relationship
	30%	672.24	73	<0.01 (<0.01)	<0.01	2.51	8191	0.012	significant increase	681.30	63	<0.01 (<0.01)	<0.01	1.16	8466	0.246	no relationship
	40%	615.96	74	<0.01 (<0.01)	<0.01	0.45	10439	0.653	no relationship	551.81	55	<0.01 (<0.01)	<0.01	−0.10	13791	0.922	no relationship
	50%	565.35	51	<0.01 (<0.01)	<0.01	−1.70	12530	0.088	no relationship	566.08	46	<0.01 (<0.01)	<0.01	0.22	13086	0.829	no relationship
East Falkland	5%	1309.24	58	<0.01 (<0.01)	<0.01	−11.17	99238	<0.001	**significant decline**	1302.49	86	<0.01 (<0.01)	<0.01	−23.11	99669	<0.001	**significant decline**
	10%	1237.56	65	<0.01 (<0.01)	<0.01	−15.79	194581	<0.001	**significant decline**	1299.42	52	<0.01 (<0.01)	0.01	−26.15	99625	<0.001	**significant decline**
	20%	1122.95	46	<0.01 (<0.01)	<0.01	−23.09	373176	<0.001	**significant decline**	1126.17	66	<0.01 (<0.01)	<0.01	−47.09	373462	<0.001	**significant decline**
	30%	1018.40	48	<0.01 (<0.01)	<0.01	−31.56	536951	<0.001	**significant decline**	1018.11	41	<0.01 (<0.01)	<0.01	−56.00	537586	<0.001	**significant decline**
	40%	919.81	31	<0.01 (<0.01)	<0.01	−34.46	686792	<0.001	**significant decline**	799.38	23	<0.01 (<0.01)	<0.01	−70.56	877316	<0.001	**significant decline**
	50%	831.35	43	<0.01 (<0.01)	<0.01	−39.47	824820	<0.001	**significant decline**	832.05	38	<0.01 (<0.01)	0.01	−72.93	825895	<0.001	**significant decline**
Gough	5%	758.03	243	<0.01 (<0.01)	<0.01	0.97	792	0.333	no relationship	754.03	148	<0.01 (<0.01)	<0.01	1.34	816	0.182	no relationship
	10%	701.61	160	<0.01 (<0.01)	<0.01	1.14	1560	0.256	no relationship	796.96	238	<0.01 (<0.01)	<0.01	0.88	839	0.381	no relationship
	20%	643.53	180	<0.01 (<0.01)	<0.01	−0.07	2911	0.940	no relationship	683.41	195	<0.01 (<0.01)	<0.01	0.25	3201	0.799	no relationship
	30%	588.20	132	<0.01 (<0.01)	<0.01	2.01	4207	0.044	significant increase	630.55	131	<0.01 (<0.01)	0.01	5.43	4521	<0.001	significant increase
	40%	542.28	97	<0.01 (<0.01)	<0.01	2.31	5509	0.021	significant increase	499.85	111	<0.01 (<0.01)	<0.01	4.01	7486	<0.001	significant increase
	50%	500.60	123	<0.01 (<0.01)	<0.01	0.04	6514	0.969	no relationship	522.58	116	<0.01 (<0.01)	<0.01	3.34	6999	0.001	significant increase
Île aux Cochons	5%	775.43	286	<0.01 (<0.01)	<0.01	−2.05	828	0.041	**significant decline**	824.88	218	<0.01 (<0.01)	0.01	2.35	877	0.019	significant increase
	10%	770.28	187	<0.01 (<0.01)	<0.01	−1.45	1658	0.148	no relationship	866.72	273	<0.01 (<0.01)	<0.01	−0.70	899	0.482	no relationship
	20%	710.79	163	<0.01 (<0.01)	<0.01	1.11	3224	0.266	no relationship	739.82	175	<0.01 (<0.01)	<0.01	1.70	3372	0.090	no relationship
	30%	651.28	185	<0.01 (<0.01)	<0.01	1.17	4590	0.243	no relationship	677.95	181	<0.01 (<0.01)	<0.01	2.73	4790	0.006	significant increase
	40%	594.14	175	<0.01 (<0.01)	<0.01	1.39	5831	0.164	no relationship	534.65	102	<0.01 (<0.01)	<0.01	5.81	8023	<0.001	significant increase
	50%	538.11	129	<0.01 (<0.01)	<0.01	1.01	7192	0.313	no relationship	561.23	146	<0.01 (<0.01)	<0.01	1.10	7445	0.271	no relationship
Île de l'Est	5%	902.19	325	<0.01 (<0.01)	<0.01	−1.85	1699	0.065	no relationship	940.83	327	<0.01 (<0.01)	<0.01	1.80	1812	0.073	no relationship
	10%	844.02	207	<0.01 (<0.01)	<0.01	1.73	3317	0.084	no relationship	951.07	242	<0.01 (<0.01)	<0.01	−0.95	1773	0.342	no relationship
	20%	774.10	170	<0.01 (<0.01)	<0.01	−2.41	6368	0.016	**significant decline**	826.06	139	<0.01 (<0.01)	<0.01	−0.15	6684	0.877	no relationship
	30%	711.35	174	<0.01 (<0.01)	<0.01	−1.21	9061	0.226	no relationship	761.28	146	<0.01 (<0.01)	<0.01	0.96	9738	0.335	no relationship
	40%	641.00	127	<0.01 (<0.01)	<0.01	−0.55	11733	0.579	no relationship	601.65	145	<0.01 (<0.01)	<0.01	−0.46	16020	0.642	no relationship
	50%	586.36	135	<0.01 (<0.01)	<0.01	0.53	14177	0.597	no relationship	621.83	136	<0.01 (<0.01)	<0.01	−3.25	15029	0.001	**significant decline**
Île de la Possession	5%	960.01	232	<0.01 (<0.01)	<0.01	−1.75	1937	0.081	no relationship	1028.16	255	<0.01 (<0.01)	<0.01	3.03	2093	0.002	significant increase
	10%	905.70	178	<0.01 (<0.01)	<0.01	−1.17	3824	0.243	no relationship	1012.58	287	<0.01 (<0.01)	<0.01	−0.92	2066	0.356	no relationship
	20%	834.05	163	<0.01 (<0.01)	<0.01	−1.58	7115	0.115	no relationship	898.10	203	<0.01 (<0.01)	0.01	9.31	7791	<0.001	significant increase
	30%	762.75	138	<0.01 (<0.01)	<0.01	−3.34	10439	0.001	**significant decline**	805.53	158	<0.01 (<0.01)	<0.01	3.40	11128	0.001	significant increase
	40%	690.67	133	<0.01 (<0.01)	<0.01	−0.25	13188	0.800	no relationship	643.41	131	<0.01 (<0.01)	<0.01	6.19	18272	<0.001	significant increase
	50%	627.49	111	<0.01 (<0.01)	<0.01	−3.36	15935	0.001	**significant decline**	667.35	100	<0.01 (<0.01)	<0.01	0.67	17219	0.501	no relationship
Macquarie	5%	844.67	180	<0.01 (<0.01)	<0.01	−3.24	1828	0.001	**significant decline**	869.10	187	<0.01 (<0.01)	0.01	−4.74	1970	<0.001	**significant decline**
	10%	800.79	196	<0.01 (<0.01)	0.01	−4.82	3507	<0.001	**significant decline**	890.23	291	<0.01 (<0.01)	<0.01	−2.42	1930	0.015	**significant decline**
	20%	740.47	155	<0.01 (<0.01)	0.01	−7.83	6800	<0.001	**significant decline**	758.96	167	<0.01 (<0.01)	0.01	−7.28	7324	<0.001	**significant decline**
	30%	676.15	147	<0.01 (<0.01)	<0.01	−5.92	9776	<0.001	**significant decline**	701.65	150	<0.01 (<0.01)	<0.01	−7.35	10600	<0.001	**significant decline**
	40%	612.35	105	<0.01 (<0.01)	<0.01	−6.60	12693	<0.001	**significant decline**	556.37	108	<0.01 (<0.01)	<0.01	−9.47	17506	<0.001	**significant decline**
	50%	563.06	78	<0.01 (<0.01)	0.01	−11.90	14981	<0.001	**significant decline**	582.63	106	<0.01 (<0.01)	<0.01	−10.40	16213	<0.001	**significant decline**
Marion	5%	1016.95	187	<0.01 (<0.01)	<0.01	2.37	3679	0.018	significant increase	1161.15	325	<0.01 (<0.01)	0.03	14.04	4143	<0.001	significant increase
	10%	968.83	208	<0.01 (<0.01)	<0.01	1.81	7208	0.070	no relationship	1178.72	234	<0.01 (<0.01)	0.02	12.78	4131	<0.001	significant increase
	20%	879.30	165	<0.01 (<0.01)	<0.01	2.35	13740	0.019	significant increase	997.31	193	<0.01 (<0.01)	0.03	26.80	15608	<0.001	significant increase
	30%	801.93	108	<0.01 (<0.01)	<0.01	4.66	19915	<0.001	significant increase	898.33	196	<0.01 (<0.01)	0.03	30.55	22483	<0.001	significant increase
	40%	733.65	144	<0.01 (<0.01)	<0.01	2.98	25180	0.003	significant increase	710.57	126	<0.01 (<0.01)	0.03	38.24	36632	<0.001	significant increase
	50%	662.70	115	<0.01 (<0.01)	<0.01	5.61	30506	<0.001	significant increase	739.27	96	<0.01 (<0.01)	0.02	33.00	34460	<0.001	significant increase
New Amsterdam	5%	705.24	334	<0.01 (<0.01)	0.01	1.83	539	0.069	no relationship	704.86	231	<0.01 (<0.01)	0.04	6.27	591	<0.001	significant increase
	10%	687.57	252	<0.01 (<0.01)	<0.01	0.17	1129	0.866	no relationship	763.03	330	<0.01 (<0.01)	0.02	3.41	608	0.001	significant increase
	20%	611.16	212	<0.01 (<0.01)	<0.01	−0.99	2083	0.322	no relationship	635.89	198	<0.01 (<0.01)	0.01	5.23	2224	<0.001	significant increase
	30%	576.24	191	<0.01 (<0.01)	<0.01	−1.36	3008	0.174	no relationship	592.41	179	<0.01 (<0.01)	0.03	10.53	3221	<0.001	significant increase
	40%	524.93	148	<0.01 (<0.01)	<0.01	−0.02	3933	0.985	no relationship	474.80	157	<0.01 (<0.01)	0.01	10.40	5336	<0.001	significant increase
	50%	481.16	156	<0.01 (<0.01)	<0.01	1.31	4756	0.189	no relationship	490.16	133	<0.01 (<0.01)	0.02	10.40	4964	<0.001	significant increase
Prince Edward	5%	580.48	217	<0.01 (<0.01)	0.01	−1.55	534	0.121	no relationship	665.86	250	<0.01 (<0.01)	<0.01	0.91	545	0.361	no relationship
	10%	607.98	182	<0.01 (<0.01)	<0.01	−1.21	1066	0.226	no relationship	642.32	158	<0.01 (<0.01)	<0.01	−0.02	578	0.988	no relationship
	20%	539.43	138	<0.01 (<0.01)	<0.01	−1.61	2076	0.108	no relationship	562.78	159	<0.01 (<0.01)	<0.01	0.89	2184	0.372	no relationship
	30%	498.93	125	<0.01 (<0.01)	<0.01	−1.53	3004	0.126	no relationship	520.24	115	<0.01 (<0.01)	<0.01	1.23	3055	0.218	no relationship
	40%	460.07	115	<0.01 (<0.01)	<0.01	−0.08	3758	0.934	no relationship	415.25	76	<0.01 (<0.01)	<0.01	0.15	5210	0.885	no relationship
	50%	410.29	98	<0.01 (<0.01)	<0.01	1.10	4704	0.271	no relationship	428.43	110	<0.01 (<0.01)	<0.01	−1.41	4937	0.160	no relationship
Tristan	5%	891.61	323	<0.01 (<0.01)	0.01	3.37	1054	0.001	significant increase	904.25	359	<0.01 (<0.01)	0.14	16.54	1044	<0.001	significant increase
	10%	875.35	240	<0.01 (<0.01)	0.02	6.68	2065	<0.001	significant increase	934.57	365	<0.01 (<0.01)	0.07	10.55	1082	<0.001	significant increase
	20%	798.28	219	<0.01 (<0.01)	0.02	9.30	3954	<0.001	significant increase	795.96	239	<0.01 (<0.01)	0.09	22.41	3936	<0.001	significant increase
	30%	713.85	191	<0.01 (<0.01)	0.02	13.33	5600	<0.001	significant increase	725.15	178	<0.01 (<0.01)	0.08	29.22	5748	<0.001	significant increase
	40%	644.14	143	<0.01 (<0.01)	0.03	15.41	7256	<0.001	significant increase	569.73	117	<0.01 (<0.01)	0.08	33.95	9410	<0.001	significant increase
	50%	589.86	151	<0.01 (<0.01)	0.02	15.13	8652	<0.001	significant increase	594.82	120	<0.01 (<0.01)	0.09	35.53	8870	<0.001	significant increase
West Falkland	5%	1269.24	92	<0.01 (<0.01)	<0.01	0.15	68817	0.880	no relationship	1266.88	25	<0.01 (<0.01)	<0.01	−7.31	69117	<0.001	**significant decline**
	10%	1208.43	69	<0.01 (<0.01)	<0.01	0.77	135157	0.440	no relationship	1270.20	67	<0.01 (<0.01)	<0.01	−12.14	69508	<0.001	**significant decline**
	20%	1092.33	29	<0.01 (<0.01)	<0.01	−1.10	259057	0.273	no relationship	1095.28	32	<0.01 (<0.01)	<0.01	−19.26	260415	<0.001	**significant decline**
	30%	990.85	32	<0.01 (<0.01)	<0.01	−5.12	372686	<0.001	**significant decline**	993.31	24	<0.01 (<0.01)	<0.01	−21.75	373997	<0.001	**significant decline**
	40%	894.70	20	<0.01 (<0.01)	<0.01	−3.71	477632	<0.001	**significant decline**	778.86	18	<0.01 (<0.01)	<0.01	−28.85	611262	<0.001	**significant decline**
	50%	809.08	23	<0.01 (<0.01)	<0.01	−9.26	572741	<0.001	**significant decline**	812.19	28	<0.01 (<0.01)	<0.01	−30.00	574233	<0.001	**significant decline**
Quantile regressions, for the median (0.5) quantile, were used to test the relationships between day and night gap−fill prediction errors (absolute difference between observed and predicted temperatures) and support (the number of spatially and temporally neighboring observations, used to estimate the missing value). The mean and minimum number of support cells with observed LST data used to calculate gap-fill predictions for each validation scenario are reported.																	
