# Supplementary material for: High resolution temperature data for ecological research and management on the Southern Ocean Islands
Source: Sci Data. 2018 Sep 4;5:180177. doi: 10.1038/sdata.2018.177 (PMC6122169; doi:10.1038/sdata.2018.177)
Supplement: Supplementary File 1 [file sdata2018177-s2.docx]

**Supplementary File 1.**

Table of Contents

Table S1 2

Global Moran’s I test outcomes for spatial autocorrelation in the number of missing land surface temperature observations per spatial cell in the remote-sensed MODIS data for the Southern Ocean Islands.

Table S2 3

Binomial Generalized Linear Model outcomes for the relationship between the presence of missing observations in remote-sensed MODIS data and spatio-temporal variables.

Table S3 4

Site information for the placement of data-loggers (Thermochron iButtons), measuring soil temperatures along an elevational transect on Marion Island from May 2002 to May 2013.

Table S4 5

Global Moran’s I test outcomes for spatial autocorrelation in the frequency of gap-fill prediction errors greater than 1 °C across 13 Southern Ocean Islands.

Table S5 6

Negative binomial Generalized Linear Model outcomes for the relationship between the frequency of gap-fill prediction errors greater than 1 °C per spatial cell, across 13 Southern Ocean Islands, and spatio-temporal variables.

Figure S1 7

Linear quantile regressions for the relationships between gap-fill prediction errors and support.

Table S1: Global Moran’s *I* test outcomes for spatial autocorrelation in the number of missing land surface temperature observations per spatial cell in the remote-sensed MODIS data, for the Southern Ocean Islands.

| **Island** | **Photoperiod** | **Moran's *I*** | **Variance** | ***P*** |  |  |
| --- | --- | --- | --- | --- | --- | --- |
| Antipodes | day | 0.69 | 0.06 | 0.002 | *** |  |
| Antipodes | night | 0.38 | 0.05 | 0.028 | *** |  |
| Auckland | day | 0.70 | <0.01 | <0.001 | *** |  |
| Auckland | night | 0.67 | <0.01 | <0.001 | *** |  |
| Bouvetøya | day | 0.92 | 0.01 | <0.001 | *** |  |
| Bouvetøya | night | 0.88 | 0.01 | <0.001 | *** |  |
| Campbell | day | 0.90 | 0.01 | <0.001 | *** |  |
| Campbell | night | 1.00 | <0.01 | <0.001 | *** |  |
| East Falkland | day | 0.63 | <0.01 | <0.001 | *** |  |
| East Falkland | night | 0.70 | <0.01 | <0.001 | *** |  |
| **Gough** | day | 0.16 | 0.01 | 0.068 |  |  |
| **Gough** | night | 0.02 | 0.01 | 0.400 |  |  |
| Heard | day | 0.96 | <0.01 | <0.001 | *** |  |
| Heard | night | 0.98 | <0.01 | <0.001 | *** |  |
| Île aux Cochons | day | 0.91 | 0.02 | <0.001 | *** |  |
| Île aux Cochons | night | 0.14 | 0.01 | 0.027 | *** |  |
| Île de l'Est | day | 0.94 | 0.01 | <0.001 | *** |  |
| Île de l'Est | night | 0.52 | 0.01 | <0.001 | *** |  |
| Île de la Possession | day | 0.85 | 0.01 | <0.001 | *** |  |
| Île de la Possession | night | 0.15 | 0.01 | 0.024 | *** |  |
| **Inaccessible** | day | 0.13 | 0.06 | 0.235 |  |  |
| **Inaccessible** | night | 0.09 | 0.06 | 0.290 |  |  |
| Kerguelen | day | 0.98 | <0.01 | <0.001 | *** |  |
| Kerguelen | night | 0.96 | <0.01 | <0.001 | *** |  |
| Macquarie | day | 0.43 | 0.01 | <0.001 | *** |  |
| **Macquarie** | night | 0.08 | <0.01 | 0.104 |  |  |
| Marion | day | 0.97 | <0.01 | <0.001 | *** |  |
| Marion | night | 0.84 | <0.01 | <0.001 | *** |  |
| **McDonald** | day | 0.78 | 0.40 | 0.053 |  |  |
| McDonald | night | 0.91 | 0.40 | 0.032 | *** |  |
| New Amsterdam | day | 0.92 | 0.02 | <0.001 | *** |  |
| New Amsterdam | night | 0.86 | 0.01 | <0.001 | *** |  |
| Prince Edward | day | 0.66 | 0.02 | <0.001 | *** |  |
| **Prince Edward** | night | -0.02 | 0.01 | 0.512 |  |  |
| South Georgia | day | 0.97 | <0.01 | <0.001 | *** |  |
| South Georgia | night | 0.75 | <0.01 | <0.001 | *** |  |
| Tristan da Cunha | day | 0.34 | 0.01 | 0.000 | *** |  |
| Tristan da Cunha | night | 0.36 | 0.01 | 0.000 | *** |  |
| West Falkland | day | 0.62 | 0.00 | 0.000 | *** |  |
| West Falkland | night | 0.77 | 0.00 | 0.000 | *** |  |
| **Summary** | **No. datasets with significant spatial autocorrelation: 33 (78.6%)**  **No. islands with significant spatial autocorrelation: 18 (85.7%)** | | | | | |

Table S2: Binomial Generalized Linear Model outcomes for the relationship between the presence of missing observations in MODIS remote-sensed LST data and spatio-temporal variables (photoperiod (day/night), season and elevation); *n*= 16,440,660, P < 0.05 (*)

| **Predictor** | | **Model**  **Coeff.** | **SE** | ***z*** | ***P*** |  |
| --- | --- | --- | --- | --- | --- | --- |
| **Photoperiod** | Night | -1.48 | <0.01 | -773.16 | <0.001 | * |
| **Month** | Autumn | -0.16 | <0.01 | -65.96 | <0.001 | * |
|  | Winter | <0.01 | <0.01 | -1.79 | 0.073 |  |
|  | Spring | 0.42 | <0.01 | 186.80 | <0.001 | * |
| **Elevation** |  | <0.01 | <0.01 | 819.25 | <0.001 | * |

Table S3: Site information for the placement of data-loggers (Thermochron iButtons), measuring soil temperatures along an elevational transect on Marion Island from May 2002 to May 2013. Monthly soil temperatures (mean & S.D.) per site presented in Supplementary File 4.

| **Site Name** | **Latitude** | **Longitude** | **Elevation (m)** | **Site Notes** |
| --- | --- | --- | --- | --- |
| Site1_0m | -46.87695 | 37.86058 | 7 | Boulder’s Beach |
| Site2_100m | -46.87672 | 37.84072 | 91 |  |
| Site3_200m | -46.88296 | 37.82254 | 189 |  |
| Site4_300m | -46.88313 | 37.81153 | 277 |  |
| Site5_400m | -46.88909 | 37.79898 | 380 | Tafelberg |
| Site6_500m | -46.89275 | 37.79052 | 490 | First Red site 1 |
| Site7_600m | -46.89447 | 37.7875 | 581 | First Red site 2 |
| Site8_700m | -46.89749 | 37.77982 | 705 |  |
| Site9_800m | -46.89803 | 37.77475 | 754 | Katedraal hut or Ned’s kop, depending on site accessibility |

Table S4: Global Moran’s *I* test outcomes for spatial autocorrelation in the frequency of gap-fill prediction errors greater than 1 °C across 13 Southern Ocean Islands. Error was calculated as the absolute difference between the observed and predicted land surface temperatures, from the 10% random point knockout validation scenarios, where 10% of the observed values were randomly deleted prior to gap-fill.

| **Island** | **Photoperiod** | **Moran's *I*** | **Variance** | ***P*** |  |  |
| --- | --- | --- | --- | --- | --- | --- |
| Auckland | Day | 0.14 | <0.01 | <0.001 | *** |  |
| Auckland | Night | 0.51 | <0.01 | <0.001 | *** |  |
| Campbell | Day | 0.33 | <0.01 | <0.001 | *** |  |
| Campbell | Night | 0.55 | <0.01 | <0.001 | *** |  |
| East Falkland | Day | 0.36 | <0.01 | <0.001 | *** |  |
| East Falkland | Night | 0.58 | <0.01 | <0.001 | *** |  |
| Gough | Day | 0.27 | 0.01 | 0.011 | * |  |
| Gough | Night | 0.24 | 0.01 | 0.022 | * |  |
| Île aux Cochons | Day | -0.03 | 0.02 | 0.557 |  |  |
| Île aux Cochons | Night | 0.25 | 0.02 | 0.020 | * |  |
| Île de l'Est | Day | 0.10 | <0.01 | 0.124 |  |  |
| Île de l'Est | Night | 0.30 | <0.01 | <0.001 | *** |  |
| Île de la Possession | Day | 0.19 | <0.01 | 0.011 | * |  |
| Île de la Possession | Night | 0.36 | <0.01 | <0.001 | *** |  |
| Macquarie | Day | -0.02 | <0.01 | 0.580 |  |  |
| Macquarie | Night | 0.43 | <0.01 | <0.001 | *** |  |
| Marion | Day | 0.27 | <0.01 | <0.001 | *** |  |
| Marion | Night | 0.64 | <0.01 | <0.001 | *** |  |
| New Amsterdam | Day | 0.22 | 0.02 | 0.061 |  |  |
| New Amsterdam | Night | 0.48 | 0.02 | <0.001 | *** |  |
| Prince Edward | Day | -0.14 | 0.02 | 0.805 |  |  |
| Prince Edward | Night | 0.26 | 0.02 | 0.032 | * |  |
| Tristan da Cunha | Day | 0.22 | 0.01 | 0.019 | * |  |
| Tristan da Cunha | Night | 0.15 | 0.01 | 0.069 |  |  |
| West Falkland | Day | 0.33 | <0.01 | <0.001 | *** |  |
| West Falkland | Night | 0.54 | <0.01 | <0.001 | *** |  |
| **Summary** | **No. datasets with significant spatial autocorrelation: 20 (76.7%)**  **No. islands with significant spatial autocorrelation: 13 (100%)** | | | | | |

Table S5: Negative binomial Generalized Linear Model outcomes for the relationship between the frequency of gap-fill errors greater than 1 °C per spatial cell across 13 Southern Ocean Islands, and spatio-temporal variables (photoperiod (day/night) and elevation). Error was calculated as the absolute difference between the observed and predicted land surface temperatures, from the 10% random point knockout validation scenarios, where 10% of the observed LST values were randomly deleted prior to gap-fill; n= 47,088; θ= 6.07; *P* < 0.05 (*)

| **Predictor** | | **Model**  **Coeff.** | **SE** | ***z*** | ***P*** |  |
| --- | --- | --- | --- | --- | --- | --- |
| **Photoperiod** | Night | -0.77 | 0.01 | -132.70 | <0.001 | * |
| **Elevation** |  | <0.01 | <0.01 | 72.99 | <0.001 | * |


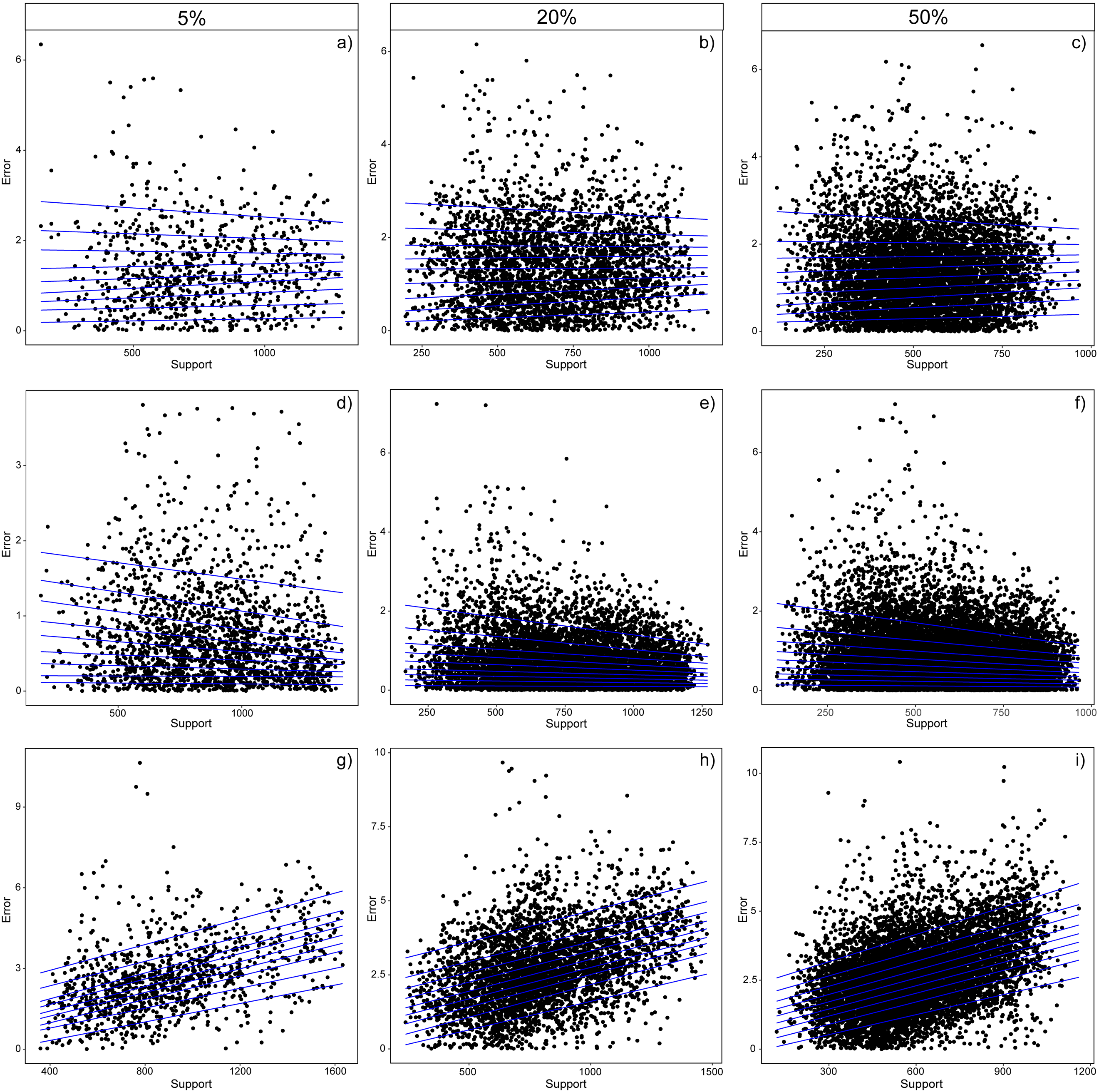


Figure S1: Linear quantile regressions for the relationship between gap-fill prediction error and support (i.e. the number of spatially and temporally neighboring cells with observed land surface temperatures (LSTs) used to predict the missing value). Gap-fill prediction error is the absolute difference between the observed and predicted LSTs. The 5, 20 and 50% cluster validation scenarios of nighttime temperatures from Gough (a, b, c), Macquarie (d, e, f) and Tristan da Cunha (g, h, i) islands are used as examples (see Table 3). Quantile regressions for the 0.1, 0.2, 0.3, 0.4, 0.5, 0.6, 0.7, 0.8 and 0.9 quantiles displayed.
